# Exploring the Science behind *Bifidobacterium breve* M-16V in Infant Health

**DOI:** 10.3390/nu11081724

**Published:** 2019-07-25

**Authors:** Chyn Boon Wong, Noriyuki Iwabuchi, Jin-zhong Xiao

**Affiliations:** 1 Next Generation Science Institute, Morinaga Milk Industry Co., Ltd., Zama, Kanagawa 252-8583, Japan; 2 Food Ingredients and Technology Institute, Morinaga Milk Industry Co., Ltd., Zama, Kanagawa 252-8583, Japan

**Keywords:** *Bifidobacterium breve* M-16V, infant health, clinical efficacy, probiotics, gut microbiota

## Abstract

Probiotics intervention has been proposed as a feasible preventative approach against adverse health-related complications in infants. Nevertheless, the umbrella concept of probiotics has led to a massive application of probiotics in a range of products for promoting infant health, for which the strain-specificity, safety and efficacy findings associated with a specific probiotics strain are not clearly defined. *Bifidobacterium breve* M-16V is a commonly used probiotic strain in infants. M-16V has been demonstrated to offer potential in protecting infants from developing the devastating necrotising enterocolitis (NEC) and allergic diseases. This review comprehends the potential beneficial effects of M-16V on infant health particularly in the prevention and treatment of premature birth complications and immune-mediated disorders in infants. Mechanistic studies supporting the use of M-16V implicated that M-16V is capable of promoting early gut microbial colonisation and may be involved in the regulation of immune balance and inflammatory response to protect high-risk infants from NEC and allergies. Summarised information on M-16V has provided conceptual proof of the use of M-16V as a potential probiotics candidate aimed at promoting infant health, particularly in the vulnerable preterm population.

## 1. Introduction

Gut microbiota has become an important aspect of human health. Gut microbes regulate host intestinal, immunological and metabolic activities through their wide array of modulatory capabilities and enzymatic armoury [1]. Recent advances in microbial research have revealed the importance of early gut microbiome for neonatal health development and disease pathologies [2]. Aberrations of infant gut microbiota—a state of altered microbial composition and functionality—are associated with adverse health-related consequences including asthma [3], necrotising enterocolitis (NEC) [4], eczema [5] and inflammatory bowel disease [6] in neonatal stage or later in life. 

Microbial ecosystem is established during the first three years of life for which a host–microbe symbiotic interaction that mutually benefits both is initiated [7]. It has been implicated that a number of extrinsic factors, such as gestational age, delivery mode and feeding types, are affecting the process of microbial colonisation in newborns [7,8]. Initial neonatal gut microbial colonisation represents a crucial window of opportunity for shaping a healthy gastrointestinal tract and immune system [9], and positive modulation of gut microbiota during this critical period could be an effective preventative approach against immune-mediated and microbiome-related disease pathologies. Consequently, probiotics intervention is receiving significant attention as a non-invasive attempt to optimize the infant microbiota as a means to improve health or prevent disease. 

Probiotics are defined as “live microorganisms, which when administered in adequate amounts, confer a health benefit on the host” [10]. Studies over the last decade have demonstrated that probiotics supplementation could promote gut microbial colonisation and prevent or treat diseases in infants [11,12]. These reports have led to a massive application of probiotics in a range of products including foods, infant formula, dietary supplements, and pharmaceutical products for promoting infant health. Nevertheless, many of the marketed probiotic products encompass limited well-consolidated regulatory oversight and a lack of human substantiation of efficacy [13]. Moreover, the safety and effects of probiotics in the vulnerable preterm population remain relatively limited and inconsistent [14,15]. Therefore, a detailed review of the scientific basis of a specific probiotic strain has emerged as an important aspect for an optimised selection of suitable probiotic candidates for use in infants. 

*Bifidobacterium breve* M-16V (designated as M-16V) is a commonly used probiotic strain in infants for modulation of gut microbiota as a means to support healthy growth and promote health. Some evidence suggests that M-16V can stimulate bifidobacterial colonisation, alleviate allergic disorders and protect premature infants against NEC. Nevertheless, despite its nutritional and medicinal benefits, a comprehensive review of its specific clinical effects for infant health is still lacking. In this review, we discuss the effects of probiotic administration on infant health, with specific attention to the probiotic strain M-16V. We conducted a systematic survey for publications related to M-16V using the databases including MEDLINE [16], EMBase [17], medical journal web [18] and JDreamIII [19] from inception to 12 May 2019. Search terms were: M-16V OR M16V and the languages used were English and Japanese. A total of 60 articles including in vitro, preclinical and clinical studies were extracted (two review and 58 original articles). Among them, 31 were on the single strain of M-16V, five were on the probiotics mixture with other strains, and 24 were on synbiotics (Supplementary Tables S1 and S2). Herein, we summarise the significant effects of M-16V on premature birth complications and allergic disorders from the most relevant in vitro, animal and clinical studies. We believed that improved understanding of the role of M-16V in governing development of healthy gut microbiota during early life would inform rational therapeutic application of probiotics aimed at promoting infant health, especially in the vulnerable preterm population, and ultimately preventing chronic diseases later in life. 

## 2. Probiotics for Infant Health 

Probiotics intervention has gained overwhelming popularity over the last two decades as a potential nutritional supplementation approach to promote and maintain a healthy gut milieu and protect against dysbiosis in early life [20]. Accumulating evidence suggests that manipulation of the microbiota with the use of probiotics at an early stage may lead to an appropriate microbial colonisation and could have long-lasting impacts on child and adult health [21]. Probiotics that have been commonly given to neonates and infants include species of *Bifidobacterium* and *Lactobacillus*. Among them, *Bifidobacterium* is thought to be a keystone taxon in infant gut microbiota that plays a vital role in regulating immunological and physiological functions [22].

Bifidobacterial species have been isolated from the gastrointestinal tract of humans and animals as well as a few that have been isolated from human vagina, oral cavity, breast milk, sewage and foods, and could be categorised into two major groups; bifidobacterial species of human origins as human-residential bifidobacteria (HRB), whereas other species which are the natural inhabitants of animals or environment as non-HRB [23]. It has been demonstrated that bifidobacterial species of different residential origins display different levels of adaptability and functionality in the infant gut [23]. Of note, *B. longum* subsp. *infantis* (*B. infantis*), *B. longum* subsp. *longum* (*B. longum*), *B. bifidum*, and *B. breve*, which are frequently isolated from infant intestines and are referred to as infant-type HRB [23,24], have a large repertoire of genes for the utilisation of human milk oligosaccharides (HMOs) [25,26]. Studies have reported that infant-type HRB are capable of utilising HMOs with different metabolic pathways and degrees of degradation, highly compatible with human breast milk and tolerant to lysozyme [25,27], demonstrating how well adapted they are to the transmission routes and growth conditions in the infant gut. In fact, infant-type HRB have been shown to be the exclusive members of healthy breastfed infants [28,29], while formula-fed infants are also colonised with species that are commonly isolated from adult intestines (adult-type HRB) such as *B. adolescentis* and *B. pseudocatenulatum* [30], implying the strains of infant-type HRB could be better probiotic candidates for infant use. 

Several studies have demonstrated the use of infant-type HRB, including the strains of *B. breve* [31], *B. longum* [32], *B. infantis* [33] and *B. bifidum* [34], as probiotics for therapeutic purposes in neonates and infants. Administration of infant-type HRB probiotic strains in the first stage of life may result in the prevention of NEC and reduction in the risk as well as treatment of infectious and atopic disease [11,12]. Despite the promise, questions and concerns have been raised about the safety and clinical efficacy of probiotics administration, especially if the product is destined for use in infants. It is increasingly apparent that not all probiotics are equally safe, and the effects demonstrated with one strain cannot be extrapolated to another strain, even if they belong to the same species [35]. Of note, among many infant-type HRB probiotic strains that have been studied, M-16V possesses a proven track record of safety and a number of beneficial attributes that make it an attractive probiotic candidate for infant use. The following paragraphs will review the safety and specific health benefits of M-16V in infants within the field that seek to provide rigorous preclinical characterisation and substantial clinical evidence of M-16V for successful probiotics selection. 

## 3. *Bifidobacterium breve* M-16V as Infant Probiotic

### 3.1. Origin and Characteristics

M-16V was originated from the gut of an infant in 1963 and was first commercially available in Japan in 1976 with the launch of Vinelac dietary supplement. In 1982, M-16V was added to a growing-up powdered formula called Yochien-Jidai in Japan and has since been incorporated in several other products including term and preterm infant formula. 

M-16V is a non-motile, non-spore forming, rod-shaped anaerobic Gram-positive bacterium. It was identified as *B. breve* based on morphological, physiological and genetic characteristics. M-16V is highly accessible to human gastrointestinal tract with strong adherence activity [36]. In addition, lyophilised powder of M-16V manufactured by Morinaga Milk Industry Co., Ltd. possesses excellent stability during storage and high survivability in finished products such as powdered formula until consumption [37].

### 3.2. Safety

M-16V is well-evaluated for safety and has met the safety standards regulated by the Food and Agriculture Organization of the United Nations/World Health Organization (FAO/WHO) guidelines for the evaluation of microbes for probiotics use in foods [38]. In 2013, M-16V attained not only FDA-Notified Generally Recognized as Safe (GRAS) status for food uses (GRN No., 453) [39], but also GRAS status for infants (GRN No., 454) [40]. In addition, in 2016, M-16V has been included in the list of authorised probiotic strains for infant’s food in China, in which M-16V is the only infant-type HRB strain among the nine strains in the list [41]. To date, there has been broad use of M-16V in low birth weight infants to reduce the risk of preterm birth complications in more than 120 neonatal intensive care units (NICU) in affiliated hospitals in Japan, Australia, New Zealand and Singapore [42,43,44].

Comprehensive safety evaluation of M-16V, which includes functional, genomic, and in vivo analyses, demonstrated that M-16V is a non-pathogenic, non-toxigenic, non-haemolytic and non-antibiotic resistant probiotic bacterium that does not contain any plasmids and does not display harmful metabolic activities [36,40,45,46]. M-16V produces L-lactic acid but no D-lactic acid. In addition, M-16V was reported to possess conjugated bile salt hydrolytic activity [36]. M-16V was able to hydrolyse conjugated bile acids taurocholic and glycocholic acid to the primary bile acid (cholic acid) and glycochenodeoxycholic and taurochenodeoxycholic acid to chenodeoxycholic acid, while the production of hepatotoxic and carcinogenic secondary bile acids (deoxycholic and lithocholic acid) was not detected upon complete biotransformation of bile salts [47]. These results resolve the concern about the safety of administering a secondary bile acids-producing bacterium. 

Studies on acute and chronic toxicological features of M-16V revealed that both single and repeated oral administration of M-16V did not cause death and any toxic symptoms in a rat model [45]. For instance, groups of 10 male and 10 female three-week-old Crj:CD (SD) rats were orally administered with a single dose of M-16V at 6000 mg/kg (1.4 × 10^12^ CFU/kg) or 3000 mg/kg (6.9 × 10^11^ CFU/kg) and examined for acute toxic symptoms for 14 days. There were no gross abnormalities or histopathological findings attributable to the treatment in all organs throughout the test period, although slightly lower body weight was observed in male rats administered a high dose of M-16V as compared to the control on days 8 and 10. Furthermore, oral administration of M-16V with a 90-day repeated dose (2.3 × 10^11^ CFU/kg/day) to five-week-old Crj:CD (SD) IGS rats revealed no adverse effects attributed to M-16V during the study period. M-16V induced no significant histopathological changes in all organs examined. These findings demonstrate the absence of acute and chronic toxicity by consumption of M-16V. Additional in vitro tests showed that M-16V did not possess mucin degradation ability [48]. Taken together, these studies support that M-16V is safe for use as a probiotic in humans.

## 4. Effects of M-16V on Premature Birth Complications

Prematurity, prolonged hospitalisation, immunodeficiency, antibiotics use and delayed enteral feeding are challenging ways to begin life for preterm infants [49]. Premature infants are at elevated risk to develop multiple health comorbidities; one of which is the devastating necrotising enterocolitis (NEC) [50]. It is a major cause of morbidity and mortality in extremely preterm infants that is associated with severe sepsis and intestinal perforation [51]. Although the exact aetiology and pathogenesis of NEC remain elusive, perturbation of the gut microbiota, leading to a hyperinflammatory response, appears to be a key factor that predisposes neonates to NEC [52]. Premature infants often present with an immature gut and exhibit delayed gut colonisation with beneficial commensal bacteria such as *Bifidobacterium* and *Bacteroides*, where instead they are more susceptible to colonisation by *Enterobacteriaceae* and *Enterococcus* [53,54]. Moreover, the use of antibiotics in premature and low birth weight infants disturbs the colonisation patterns of *Bifidobacterium* and shifts the gut microbial composition toward a high abundance of Proteobacteria, with a decreased in the overall diversity of the infant’s gut microbiota [55,56,57]. To this end, the neonatal period has; therefore, emerged as an opportune time for preventive M-16V probiotics intervention to promote bifidobacterial colonisation, facilitate the development of gut mucosal immune system and improve infant health.

### 4.1. Preclinical Studies

Several animal studies have demonstrated the potential role of M-16V in improving the maturation of intestinal immune system and promoting bifidobacterial colonisation during early infancy. In a neonatal Lewis rat model, oral supplementation with M-16V (4.5 × 10^8^ CFU/100 g of body weight/day; *n* = 8) during suckling period (days 6 to 18) showed potential in enhancing the homing process of naïve T cells to the mesenteric lymph nodes (MLN) and the retention of activated T cells in the intraepithelial (IEL) compartment [58]. The control group (*n* = 8) was administered with a matched volume of mineral water. Administration of M-16V increased the proportion of cells bearing toll-like receptor 4 (TLR4) in the MLN and IEL compartments, and enhanced the percentage of the integrin αEβ7+ and CD62L+ cells in the MLN and that of the integrin αEβ7+ cells in the IEL, as compared to the control. However, M-16V did not exert a systemic immune-enhancing effect in which the proportions of the main lymphocyte subset in spleen were not significantly affected by M-16V. In addition, M-16V induced no harmful effects on the rats wherein no significant differences were observed in the growth curve of the control and M-16V groups. Administration of M-16V significantly increased the levels of intestinal immunoglobulin A (IgA) as compared to the control, indicating M-16V could also strengthen the humoral intestinal immune response. 

Furthermore, M-16V has also been reported to be able to regulate immune responses and appear to exert anti-inflammatory effects in rats at different developmental periods [59,60]. Oral administration of M-16V (5 × 10^8^ CFU/day) to F344/Du rats significantly reduced the expression of inflammatory molecules during the newborn period (days 1 to 14) and regulated the expression of co-stimulatory molecules during the weaning period (days 21 to 34) [60]. In addition, the numbers of *Bifidobacterium* were also significantly increased in both the caecum and colon during the newborn period but not during weaning, as compared to the control groups [60]. 

Similarly, significant improvements in inflammatory conditions were also observed in DSS-induced colitis F344/N rats administered with M-16V (2.5 × 10^9^ CFU/day) during weaning period (from postnatal days 21 to 34), as compared to the control rats. M-16V showed potential in altering systemic T-cell immune functions and suppressing inflammatory responses in colitis rats during the weaning period [59]. Taken together, these preclinical studies imply that supplementation with M-16V may aid in the development of intestinal immunity and prevention of intestinal inflammation during early infancy. 

### 4.2. Clinical Studies

Multiple implementation cohort studies have demonstrated the potential effect of early administration of M-16V in improving bifidobacterial colonisation in preterm infants (gestation < 33 weeks) [42,61] and low birth weight infants (<2250 g) [32,42,62,63]. Earlier detection and longer maintenance of a bifidobacteria-dominant gut microbiome were observed in M-16V-supplemented infants. For instance, in a randomised, double-blind, placebo-controlled trial involving 159 preterm neonates (gestation < 33 weeks) ready to commence or on feeds for <12 h, supplementation of M-16V (3 × 10^9^ CFU/day) for three weeks significantly increased the levels of faecal *B. breve* as compared to placebo control where the *B. breve* counts were below detection level [42]. M-16V supplement was well-tolerated by all enrolled preterm neonates with no adverse effects including probiotic sepsis and deaths. These findings suggest that M-16V is a suitable probiotic strain for routine use in preterm neonates to promote the acquisition of beneficial commensal bacteria. 

Another randomised, placebo-controlled trial involving 30 preterm low birth weight infants, with mean gestation 32.8 weeks and birth weight 1486 g, also revealed a positive effect of M-16V on early gut colonisation with commensal *Bifidobacterium* spp. [63]. The subjects were randomly divided into three groups; (A) subjects received M-16V supplementation within several hours (mean: 7.2 h) of birth, (B) subjects received M-16V supplementation >24 h (mean: 36.5 h) after birth, and (C) subjects who were fed normally without M-16V supplementation as control group. Intragastrical administration of M-16V (1.6 × 10^8^ CFU in 0.5 mL of 5% glucose sterile distilled water, twice daily) until the subjects were discharged from the hospital remedied the delayed bifidobacterial colonisation in both groups A and B, while no *Bifidobacterium* was detected in eight out of ten infants in group C during the observation period of seven weeks [63]. Notably, a significant earlier detection of bifidobacteria and a significant decrease in the cell numbers of *Enterobacteriaceae* were observed at two weeks after birth in infants administered with M-16V within several hours of birth (group A), indicating timing of administration of M-16V is highly important for which the earlier the administration of M-16V to preterm low birth weight infants, the better the effects of M-16V in promoting the colonisation of bifidobacteria and reducing the susceptibility to colonisation by potentially harmful bacteria.

A comparative, non-randomised controlled, prospective trial involving 44 low birth weight infants (body weight 1000–2000 g), who were ready for feeds within seven days of birth, administered with either single strain of M-16V (5 × 10^8^ CFU/day) or probiotics mixture containing three bifidobacterial strains, M-16V, *B. infantis* M-63, and *B. longum* BB536 (5 × 10^8^ CFU/day of each strain), for six weeks has also revealed a significant increase in the detection rates and cell numbers of bifidobacteria in the faeces [32]. Notably, administration of the three-species probiotics mixture resulted in an earlier formation of a bifidobacteria-dominant microbiota and a significantly lower level of *Enterobacteriaceae* than those administered with M-16V alone [32]. It was noted that not only the total cell numbers of bifidobacteria but also the cell numbers of M-16V was higher in infants administered with the three-strain probiotics mixture than those administered with M-16V alone. This study suggests that M-16V may act synergistically and cooperatively with other *Bifidobacterium* strains to confer a more remarkable beneficial effect in premature infants. Nevertheless, the comparison between probiotics and control groups from the different timeline in this trial may introduce bias that tends to compromise the efficacy of M-16V and is likely to result in unfair comparisons. Additionally, two comparable pilot studies involving ten very low birth weight premature infants (<1250 g) administered with either M-16V or *B. longum* at a dose of 5 × 10^8^ CFU/day for eight weeks have also suggested a potential capability of M-16V to colonise in the premature gut [62,64]. Supplementation with M-16V had a longer colonisation rate than those with *B. longum*, for which, while M-16V was found to colonise the premature gut as early as week two after birth and remain dominant, the administered strain of *B. longum* was not detected from week six after birth [62,64]. Collectively, these data have exemplified that M-16V is potentially beneficial at promoting early colonisation of bifidobacteria and may; therefore, support healthy growth in premature infants. 

Furthermore, M-16V has also been evaluated for the preventive effects on NEC, death and late-onset sepsis in premature infants; however, the clinical findings are not conclusive [43,44,65]. The first evidence of the potential preventive effects of M-16V on NEC came from a non-randomised clinical trial involving 338 infants (220 extremely low birth weight (ELBW) and 118 very low birth weight (VLBW) infants) receiving M-16V supplementation (1 × 10^9^ CFU/day in raw breast milk or formula milk) started within several hours (mean 7.2 h) after birth and continued until discharged from NICU, and 226 infants (101 ELBW and 125 VLBW infants) as a historical control [44]. The study revealed that administration of M-16V was potentially effective at reducing the incidence of NEC in ELBW and VLBW infants as compared to that in the historical control group. A significant reduction in morbidity and mortality rate, as well as the mortality due to infection, was also observed in ELBW and VLBW infants receiving M-16V supplementation [44]. These encouraging results have suggested a potential role of M-16V in protecting premature infants from NEC and infection. However, the use of historical control from another timeline in this trial may introduce bias that tends to compromise the efficacy of M-16V and is likely to result in unfair comparisons. 

More recently, M-16V was reported to be associated with decreased incidence of “NEC ≥ Stage II” and “NEC ≥ Stage II or all-cause mortality” in preterm neonates <34 weeks [43]. The study was a retrospective cohort study involving 835 preterm neonates as historical control and 920 preterm neonates receiving M-16V routine probiotics supplementation (3 × 10^9^ CFU/day in 1.5 mL breast milk or sterile water) started when the infants were ready for enteral feeds and continued until the corrected age of 37 weeks. The initial daily dose for neonates <28 weeks was 1.5 × 10^9^ CFU/day until reaching feeds of 50 mL/kg/day. It was noted that M-16V significantly lowered the incidence of NEC in preterm VLBW neonates born <34 weeks, while the incidence of NEC was lower but not statistically significant in those born <28 weeks, although the small sample size used [43]. Despite the encouraging results, the trial may introduce potential bias in comparisons with the historical control drawn from another timeline. 

In addition, a recent strain-specific systematic review revealed that the significant efficacy of M-16V to reduce the risk of NEC remains controversial [65]. It was concluded that current evidence is limited regarding the potential of M-16V as a probiotic for preterm neonates, albeit the meta-analysis of non-randomised controlled trials showed a significant effect of M-16V intervention in NEC [65]. No significant benefits on stage ≥2 NEC, late-onset sepsis, mortality and postnatal age at full feeds were reported in the meta-analysis of randomised controlled trials. Well-designed and adequately-powered randomised controlled trials are needed for definite confirmation. Nonetheless, all clinical studies included in the systematic review have concluded that M-16V supplementation was not associated with probiotic-associated sepsis in this vulnerable population [65], suggesting the risk of developing sepsis related to M-16V administration in the setting of severe illness to be relatively low. In fact, issues on *B. breve* sepsis in immunocompromised infants [66,67] and meningitis caused by other strain of *B. breve* in preterm infants [68] have been reported. Another systematic review using a network meta-analysis approach showed that only few probiotic strains have statistically significant effects in reducing mortality, NEC, late-onset sepsis, and time until full enteral feeding [69]. M-16V was one of the many studied probiotic strains that did not show significant efficacy in preterm birth complications, reflecting a lack of adequately-powered randomised controlled trials to precisely define the clinical efficacy [69]. Further large and well-powered trials are needed to evaluate the effectiveness of M-16V in preventing NEC. 

Taken together, these clinical studies underscore the potential roles of M-16V as a promising infant probiotic that could potentially impact the incidence, morbidity and mortality associated with NEC (Table 1). 

### 4.3. Potential Mechanisms of Action

Colonisation by commensal bifidobacteria during early life is indispensable for the normal development and growth of the gastrointestinal tract, particularly for epithelial barrier function and mucosal immunity [72,73]. A high abundance of bifidobacteria may contribute to improved health status and protect premature infants from diseases [74]. In fact, instability of the microbiome and a lack of bifidobacteria have been reported to be associated with NEC [74]. Towards this end, it seems likely that M-16V may potentially reduce the risk of developing NEC in premature infants by promoting the colonisation of bifidobacteria. Additional studies have been deployed to understand the mechanisms by which M-16V potentially reduces the risk of developing NEC [60,75]. In an experimental rat model of NEC, oral administration of M-16V was found to be effective at reducing the pathological scores of NEC and promoting survivability via modulation of TLR expressions and suppression of inflammatory responses [75]. Multiple reports have suggested that functional expression of TLRs is critical in the dynamic interaction between the host epithelium and the microbiota that enables normal intestinal epithelial development and immune homeostasis [76,77,78]. Differences in the expression of TLRs may; therefore, alter a host’s response to a commensal or pathogenic microorganism [79]. Specifically, TLR4, which recognises the lipopolysaccharides of Gram-negative bacteria, was demonstrated as the key mediator in NEC development [76]. Increasing evidence suggests that NEC develops in response to an exaggerated pro-inflammatory signalling upon activation of TLR4 in the mucosa of the premature gut, leading to increased enterocyte apoptosis, mucosal injury, intestinal ischemia, and bacterial translocation [76,77,79,80]. It has indeed been demonstrated that TLR4 is expressed at higher levels in the premature infant gut than the full-term intestine [76,81]. Importantly, oral administration of M-16V to the experimental NEC rats significantly normalised the expression of TLR4, enhanced the expression of TLR2, and rectified the increased expression of pro-inflammatory cytokines, including interleukin-1β (IL-1β), IL-6 and tumour necrosis factor alpha (TNF-α) that resulted from NEC induction [75]. The superior anti-inflammatory effects of M-16V in colonic inflammation have also been demonstrated in an in vivo study using F344/Du rat pups models, wherein the expression of inflammation-related genes, including lipoprotein lipase (Lpl), glutathione peroxidase 2 (Gpx2) and lipopolysaccharide-binding protein (Lbp), was significantly reduced in the colon during the newborn period [60]. 

Furthermore, M-16V was also able to restore the tight junction barrier function by stimulating TLR2 expression and consequently protect the host against the development of NEC [75]. It has been reported that enhanced TLR2 expression by probiotics treatment could contribute to the down-regulation of TLR4 signalling that is activated by NEC [82,83]. Of note, aberrant TLR4 signalling was found to have a direct role in the breakdown of the gut barrier in NEC. Enhanced TLR4 signalling impairs mucosal repair and weakens the integrity of the gut, allowing for bacterial translocation and the downstream inflammatory response, which in aggregate lead to NEC [77]. Remarkably, M-16V showed potential in protecting the experimental NEC rats from intestinal barrier dysfunction via suppression of the NEC-induced elevated expressions of tight junction-related proteins, including ZO-1, claudin-1 and occludin [75]. 

Studies have also shown that daily M-16V supplementation may potentially facilitate the development of gut immune function and attenuate inflammation in preterm infants [58,59,70]. Administration of M-16V (1 × 10^9^ CFU in 0.5 mL of 5% glucose solution), starting several hours after birth, twice daily, was shown to be capable of significantly elevating the levels of serum transforming growth factor beta-1 (TGF-β1) and enhancing the expression of TGF-β signalling molecule Smad3, while suppressing the levels of Smad antagonist, Smad7 in 19 preterm infants (mean birth weight of 1,378 ± 365 g and mean gestational age of 31.3 ± 3.16 weeks) as compared to the control on day 28 [70]. TGF-β1 is an important immune regulatory cytokine that prevents adverse immunologic reactions in infants. It exerts potent anti-proliferative and anti-inflammatory effects by activating Smad signalling pathway that mediates cell cycle arrest and induction of apoptosis [84]. Deficiency in TGF-β1 or its receptor has been implicated in fulminant inflammatory disease that proves lethal in the first week of life [85]. The encouraging result obtained in the clinical study has; therefore, implied that M-16V may assist the development of mucosal immunity and attenuate inflammatory reactions in preterm infants through upregulation of TGF-β signalling.

In addition to interacting with the immune system, M-16V may also potentially protect premature infants against gut mucosal injury and NEC through the production of short chain fatty acids (SCFAs) that can affect the health and integrity of the intestinal epithelial and immune cells [71]. In a randomised controlled trial involving 66 premature infants (birth weight ranged from 414 to 2124 g and gestation age ranged from 23 to 36 weeks), the effects of oral administration of M-16V on faecal SCFAs were evaluated. Based on birth weight, the infants were divided into three groups: 22 extremely low birth weight infants (ELBW; <1000 g), 22 very low birth weight infants (VLBW; <1500 g), and 22 low birth weight infants (LBW; <2500 g) and within each group, the subjects were further randomly divided into M-16V-supplemented or control groups. Administration of M-16V (1.6 × 10^8^ CFU in 0.5 mL of 5% glucose sterile distilled water) at time of normal feeding, twice daily for four weeks led to an intestinal environment where the levels of butyrate was significantly decreased in ELBW and VLBW infants, while the ratio of acetate to total SCFAs was significantly increased in ELBW, VLBW, and LBW infants as compared with those of the control groups [71]. The exact significant contribution of such changes in the levels of SCFAs to premature infant health upon M-16V administration remains unclear. Although evidence is limited, higher acetate level in infants, which is often associated with a high abundance of bifidobacteria, has been reported to potentially improve intestinal immunity and promote epithelial cell barrier function [86,87]. Nevertheless, the healthy composition of an infant faecal metabolome remains understudied.

The premature gut is known to have structural and biochemical deficiencies which predispose infants to NEC. Although bacterial production of SCFAs plays an important role in the intestinal maturation and functions, it has been reported that overproduction of certain SCFA could be associated with an increased risk of NEC in premature infants [88]. Study has suggested that *Clostridium* spp., for which the abundance was higher in premature infants, may be implicated in NEC through excessive production of butyrate as a result of colonic lactose fermentation [89]. Overproduction of butyrate may cause gut mucosal injury and lead to intestinal inflammation in premature infants [90,91]. However, numerous studies have also demonstrated the importance of butyrate for colon health and its beneficial effects on intestinal inflammation and barrier integrity [92,93,94]. Further studies are warranted to resolve the contradictory roles of butyrate and to investigate the association between reduction of butyrate production by M-16V and protection against NEC.

**Figure 1:**
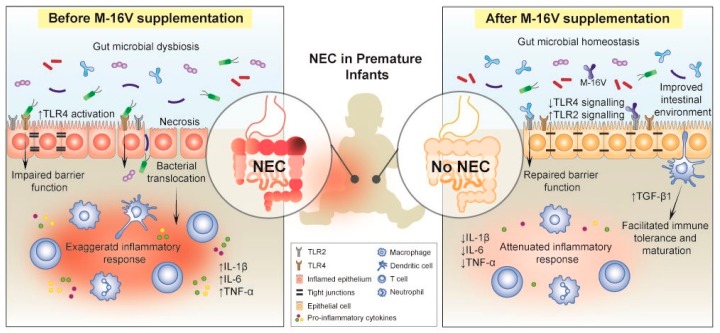
Administration of *Bifidobacterium breve* M-16V showed potential in reducing the risk of developing necrotising enterocolitis (NEC) in premature infants. M-16V stimulates the colonisation of bifidobacteria and could potentially improve the intestinal environment and gut barrier function. Additional mechanistic studies revealed that M-16V may assist the development of mucosal immunity through up-regulation of transforming growth factor-beta (TGF-β) signalling in premature infants and attenuate inflammatory reactions by modulating the expressions of toll-like receptor 2 (TLR2) and TLR4. IL-1β, interleukin-1β; IL-6, interleukin-6; TNF- α, tumour necrosis factor alpha; ↑, increased; ↓, decreased.

Taken together, the findings from both animal and clinical studies have shed lights into the potential protective mechanisms of M-16V against NEC in premature infants. It is evident that M-16V may potentially reduce the risk of developing NEC in premature infants by promoting bifidobacterial colonisation, modulating the expressions of TLRs and inflammatory responses, and aiding in the development of mucosal immunity (Figure 1).

## 5. Effects of M-16V on Allergic Disorders

The prevalence of allergic diseases in infants has increased strikingly worldwide in the past few decades [95]. While the pathogenesis of allergic diseases is likely to be multifactorial, deviations in gut colonisation during early life are possible major factors promoting abnormal postnatal immune maturation [96]. The hygiene hypothesis suggests that insufficient or aberrant microbial stimulation during the critical neonatal period may lead to an exaggerated adaptive immune response and reduced tolerance [97]. Although compelling evidence for microbiota associations with allergic disease and related conditions is emerging, a causal relation between specific bacterial taxa and the development of allergy remains unclear. Several studies have reported differences in gut microbiota composition and lower abundance of bifidobacteria and lactobacilli in the infant’s gut precede the onset of allergic manifestations [98,99]. In addition, multiple cohort studies suggested that high abundance of *Escherichia coli* or *Clostridium difficile* was associated with the development of eczema or atopy [100,101], while a low gut microbial diversity and an elevated *Enterobacteriaceae* to *Bacteroidaceae* (E/B ratio) in early infancy may contribute to the development of food allergy [102]. In this instance, a notable higher abundance of Firmicutes particularly *Clostridium* spp., *Blautia* spp., and a lower abundance of Actinobacteria in the early gut microbiota has also been described to contribute to the development of allergic diseases such as food allergy in infants [103], and type 1 diabetes in children [104]. On this basis, modulation of gut microbiota during early life through M-16V intervention has emerged as a potential measure to prevent allergic disorders in infants.

### 5.1. Preclinical Studies 

The anti-allergic capability of M-16V in allergic airways disease, food allergy, and chronic asthma has been consolidated in a number of in vitro and animal studies [105,106,107,108,109,110]. In a bacterial strains comparative study assessing the capability of a panel of six bacterial strains (M-16V, *B. infantis* NumRes251, *B. animalis* NumRes252 and NumREs253, *Lactobacillus plantarum* NumRes8 and *L. rhamnosus* NumRes6) to alleviate allergic symptoms in ovalbumin (OVA)-sensitized BALB/c mice, M-16V was identified as the most effective strain in reducing allergic response [105]. Remarkably, in contrast to the other tested bifidobacteria, only the oral treatment with M-16V significantly inhibited the airway reactivity to methacholine and reduced acute allergic skin reactions to OVA. These discrepancies emphasise that the immuno-modulatory activity of probiotic strains is highly strain-specific.

Numerous studies have also shown that a synbiotic intervention, comprising M-16V and a galacto–fructooligosaccharide (GOS/FOS) mixture, was protective against the development of symptoms of oral sensitization with whey in mice model [110]. The promising effect was confirmed in an in vivo study demonstrating the partial prevention of skin reaction due to cow’s milk allergy, following the probiotic administration in combination with specific β-lactoglobulin-derived peptides and a specific blend of short and long-chain fructo-oligosaccharides in mice [106]. Particularly, besides increasing the caecal content of propionic and butyric acid, the treatment with M-16V synbiotic formulation increased the expression of IL-22, which plays an antimicrobial role in the innate immune response and on the anti-inflammatory cytokine IL-10 in the Peyer’s patches [106].

Additional preclinical studies revealed that administration of M-16V alone (10^9^ CFU) [108], or in combination with non-digestible oligosaccharides (scFOS, lcFOS and pectin-derived acidic-oligosaccharides (AOS)) [109], could suppress pulmonary airway inflammation in murine OVA-induced chronic asthma model. M-16V treatments (both single-strain and synbiotic interventions) reduced T cell activation and mast cell degranulation, modulated expression of pattern recognition receptors, cytokines and transcription factors, and reduced airway remodelling [108,109]. More specifically, the treatments induced regulatory T cell responses in the airways by increasing IL-10 and Foxp3 transcription in lung tissue and systemically. These studies suggest that M-16V intervention, either as a single organism or as synbiotic, could be beneficial in the treatment of chronic inflammation in allergic asthma. Altogether, these findings laid the ground for the preventive and therapeutic effects of M-16V on allergic disorders. 

### 5.2. Clinical Studies

Several interventional studies suggest that M-16V could promote bifidobacterial colonisation and prevent or reduce the severity of allergic diseases, including atopic dermatitis (eczema), food allergy, allergic rhinitis and asthma [111,112,113]. In a randomised controlled trial, oral administration of M-16V significantly improved the symptoms of atopic dermatitis in infants as compared to the control group [112]. The study randomly allocated 15 infants (aged 8.6 ± 4.5 months) with atopic dermatitis who had a *Bifidobacterium*-deficit gut microbiota to receive either lyophilised powder of M-16V (*n* = 8; 5 × 10^9^ CFU/day) for one month or no M-16V supplementation as a control. It was noted that administration of M-16V was not only effective at alleviating the severity of allergic symptoms but also significantly increased the proportion of *Bifidobacterium* and decreased the levels of total aerobes in the gut microbiota of infants with atopic dermatitis [112]. Nevertheless, a significant correlation between alleviation of allergic symptoms and changes of the gut microbiota was not detected; suggesting M-16V may possess a direct immuno-modulatory effect on intestinal epithelial cells and not necessarily through the interaction with the gut microbiota.

Another clinical study involving 17 infants with cow’s milk hypersensitivity with atopic dermatitis (aged 3.1–18.5 months) has also revealed the capability of M-16V supplementation (5 × 10^9^ CFU/day for three months) to ameliorate allergic symptoms and improve gut microbiota composition [113]. The preventive effects of M-16V on allergic disorders have further been exemplified in a remarkable placebo-controlled, double-blinded and randomised trial involving 40 Italian children (mean age 9 ± 2.2 years) treated with a probiotics mixture containing M-16V (1 × 10^9^ CFU), *B. longum* BB536 (3 × 10^9^ CFU) and *B. infantis* M-63 (1 × 10^9^ CFU), for four weeks [111]. Administration of probiotics mixture protected the children against pollen-induced IgE-mediated allergic rhinitis and intermittent asthma and improved their quality of life, for which these parameters were worsened in the placebo group. This study implies that, in addition to its effectiveness as a single organism, as aforementioned, M-16V could also dampen allergic disorders when combined with other *Bifidobacterium* strains. 

More interestingly, in an open trial, administration of a probiotics mixture including M-16V during pregnancy as well as in postnatal period tied to lower the risk of developing allergic disorders in infants [114]. The study involved 130 mothers who were provided with a daily powder formulation (two sachets daily, 1 g/sachet) containing M-16V and *B. longum* BB536 (5 × 10^9^ CFU/g of each strain) one month before the expected date of delivery and postnatally to their infants (one sachet daily) for six months. Another 36 mother–infant pairs who did not receive the bifidobacterial supplementation were served as the control. Prenatal and postnatal supplementation with the bifidobacteria mixture significantly reduced the risk of developing eczema and atopic dermatitis in infants during the first 18 months of life as compared to the control group [114]. Additionally, the probiotics intervention (M-16V and *B. longum* BB536) resulted in slight changes in the gut microbial composition, wherein a significantly higher proportion of *Bacteroidetes* was observed in the microbiota of infants receiving the bifidobacteria mixture than in that of the control group at four months of age. The relative abundance of Proteobacteria was also significantly lower in mothers receiving the bifidobacteria mixture at the time of delivery than those in the control group, and was positively correlated with that of infants at four months of age. These findings implicate that supplementation with bifidobacteria mixture of M-16V and *B. longum* BB536 during pregnancy may modulate both the maternal and neonatal gut microbiota for prevention of allergies upset in infants later in life. Further studies are needed to elucidate the association between the probiotics-modulated gut microbiota and allergy development in infants. Collectively, these findings are cautiously promising with respect to the use of probiotics for the primary prevention of eczema in pregnant mothers of infants at high risk for developing allergy and in high-risk infants, as recommended in recent guidelines from the World Allergy Organization [115].

Furthermore, synbiotic intervention of M-16V has also been reported to be effective in preventing asthma-like symptoms in infants with atopic dermatitis [116]. The study was a double-blind, placebo-controlled, multicentre trial involving 90 infants with atopic dermatitis (aged <7 months) who received either an extensively hydrolysed formula containing M-16V (1.3 × 10^9^ CFU/100 mL and a GOS/FOS mixture (90%/10%; 0.8 g/100 mL) or the same formula without synbiotics for 12 weeks. The follow-up period for this trial was one year. It was noted that the synbiotic intervention significantly reduced the prevalence of frequent wheezing and/or noisy breathing apart from colds as well as the usage of asthma medication as compared to the placebo group [116]. As a result, it seems to be likely that combining M-16V with prebiotics—synbiotic intervention—could result in stronger immunomodulatory effects for prevention against allergic disorders. Collectively, these findings serve as a basis to incorporate M-16V in prebiotics-supplemented infant formula as a means to promote infant health.

Taken together, these clinical findings support the notion that administration of M-16V can be a potential prophylaxis approach to improve immune tolerance and consequently protect high-risk infants from allergic diseases (Table 2), although larger clinical trials are needed for definite confirmation.

### 5.3. Potential Mechanisms of Action

**Figure 2:**
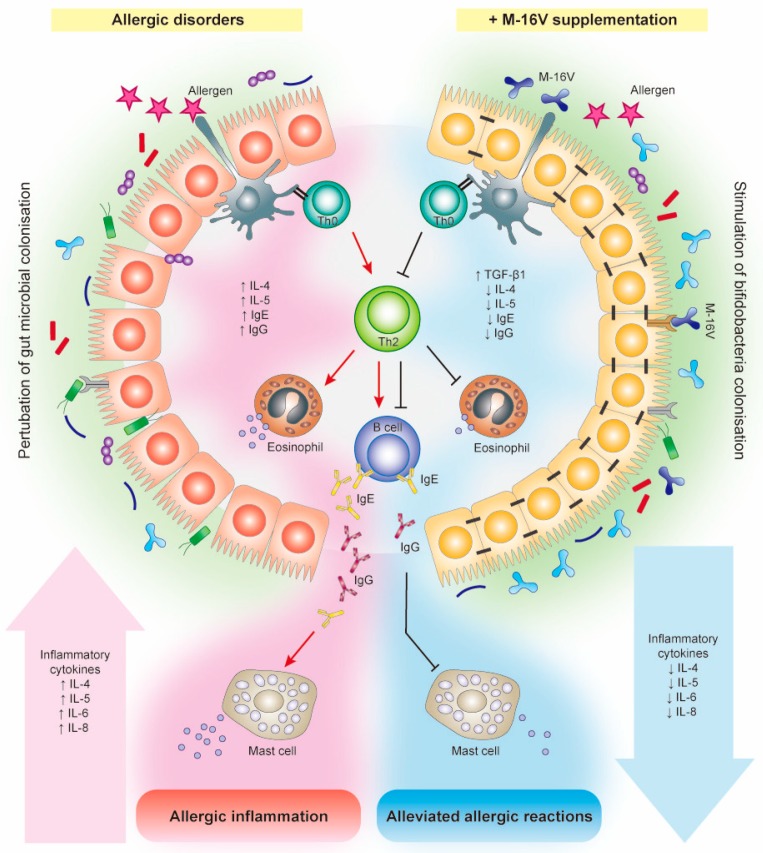
*Bifidobacterium breve* M-16V could potentially promote bifidobacterial colonisation and may prevent or reduce the severity of allergic diseases in infants. Specifically, M-16V may suppress the differentiation naïve T-helper cells (Th0) into T-helper (Th) 2 cells and the production of Th2 cytokines such as interleukin-4 (IL-4) and IL-5, and subsequently attenuate allergic inflammation by reducing the production of immunoglobulin E (IgE) and IgG1 in B cells and the release of pro-inflammatory mediators including IL-6 and IL-8. In addition, M-16V could also potentially assist immune tolerance and attenuate allergic reactions in infants through modulation of TGF-β signalling. ↓, decreased; ↑, increased.

The mechanisms through which M-16V acts to protect infants against allergic disorders are not fully understood but clearly involve the contributions from M-16V to promote bifidobacterial colonisation, modulate Th2-skewed immune response and attenuate inflammatory reactions (Figure 2). M-16V has been shown to exert immuno-regulatory effect and anti-inflammatory capability in vitro, albeit the effect on allergic reaction has not been specifically demonstrated. M-16V was reported to interact with TLR2, upregulate the expression of ubiquitin-editing enzyme A20 in porcine intestinal epithelial cells challenged with heat-killed enterotoxigenic *Escherichia coli*, and beneficially modulate the subsequent TLR4 activation by reducing the activation of MAPK and NF-κB pathways and the production of pro-inflammatory cytokines (IL-8, monocyte chemotactic protein (MCP)-1, and IL-6) [83]. Furthermore, in an experimental OVA-immunised mice model, oral administration of M-16V (5 × 10^8^ CFU/0.5 mL/day/animal) for 21 days significantly reduced the serum levels of total IgE, OVA-specific IgE and OVA-specific IgG1 and ex vivo production of IL-4 by the splenocytes, as compared to control [117]. In addition, M-16V could potentially modulate the systemic Th1/Th2 balance in vitro wherein the production of OVA-induced total IgE and IL-4 was suppressed and the secretion of IFN-γ and IL-10 was induced by M-16V in a dose-dependent manner. Nonetheless, M-16V did not induce IL-12 production. It is; therefore, suggested that M-16V may have the potential to restore Th2 skewed immune response, which was at least partially independent of the Th1 cytokine induction [117]. 

It has been suggested that the pathology of allergic disease is driven by the allergen-specific Th2 cytokines such as IL-4 and IL-5, which play a triggering role in the activation/recruitment of IgE antibody-producing B cells, mast cells and eosinophils [118,119,120]. Notably, in an OVA-allergic asthma mouse model, oral administration of M-16V (10^9^ CFU/0.4 mL/day/animal) for 17 days prevalently reduced the number of eosinophils in the bronchoalveolar lavage fluid and reduced the levels of OVA-specific IgE and IgG1 and Th2 cytokines (IL-4 and IL-5) [105]. In addition, M-16V has also been shown to potentially assist immune tolerance and attenuate allergic reactions in premature infants through modulation of TGF-β signalling [70]. Altogether, these findings provide proof of the potential of M-16V in modulating Th2 skewed allergic immune response. Further in-depth studies are required to elucidate the exact mechanisms by which M-16V prevents and ameliorates allergic disorders in infants. 

## 6. Conclusions

*Bifidobacterium breve* M-16V has emerged as a probiotic strain that exerts positive effects on infant health. With the data from in vitro animal and clinical studies, M-16V holds promise to treat adverse health-related conditions in infants, particularly the vulnerable premature populations, and possesses a proven track record of safety. Mounting evidence favours the use of M-16V as a worthy and suitable infant probiotic in early life for promoting a healthy gut microbial colonisation and maturation in premature infants and preventing the development of NEC and allergic diseases. Although the mechanistic insights supporting the use of M-16V are not robust, it has become clear that M-16V may modulate the gut microbiota, interact with TLRs and regulate inflammatory responses to reduce the risk of developing life-threatening diseases and immune-mediated disorders. Despite the promising results, many studies summarised here have multiple limitations such as potential bias in non-randomised controlled trials and small sample size. Therefore, additional well-designed randomised controlled trials with larger sample size are needed to serve as the basis for developing conclusive evidence on M-16V intervention in vulnerable preterm populations. In addition, further investigations are required for an increased understanding of the protective mechanisms of M-16V and to releasing the full potential of M-16V as a human probiotic in paediatrics.

## Figures and Tables

**Table 1:** Summary from clinical studies of the effects of M-16V on premature birth complications.

Reference	Study Design	Study Characteristics
Effect of M-16V on bifidobacterial colonisation		
Patole et al., 2014 [42]	Randomised, double-blinded, placebo-controlled	Study population: Preterm infants (<33 weeks; BW < 1500 g)
		Country: Australia
		Sample size: *n* = 159 (Probiotics: 79; Placebo: 80)
		Intervention and dose: 3 × 10^9^ CFU/day in 1.5 mL of sterile water or breast milk.
		Duration of supplementation: Supplementation started when infants were on enteral feeds for <12 h, continued till 37 weeks of corrected gestational age.
		Main outcomes: (1) Significant increase in *B. breve* faecal counts three weeks after M-16V supplementation. (2) No probiotic sepsis and death in M-16V-supplemented infants.
		Study limitations: Nil
Patole et al., 2016 [61]	Non-RCT comparative analytical study	Study population: Preterm infants (<33 weeks; BW < 1500 g)
		Country: Australia
		Sample size: *n* = 159; subjects were divided into two groups based on their gestational age: (1) SGA due to IUGR (Probiotics: 22; Placebo: 20). (2) Non-SGA (Probiotics: 55; Placebo: 56)
		Intervention and dose: 3 × 10^9^ CFU/day in 1.5 mL of sterile water or breast milk.
		Duration of supplementation: Supplementation started when infants were on enteral feeds for <12 h, continued till 37 weeks of gestational age.
		Main outcomes: (1) *B. breve* faecal counts did not differ between SGA and non-SGA infants. (2) M-16V-treated SGA infants reached full feeds earlier than SGA controls, after adjustment for age at starting feeds and gestation <28 weeks.
		Study limitations: (1) This was a comparative analytical study that relies on the results obtained from the previous study [42]. (2) Assessment of outcome could not be blinded; however, that was not expected to introduce a major bias because of the objectivity of the outcome.
Li et al., 2004 [63]	Randomised controlled trial	Study population: Preterm VLBW infants (BW < 1250 g)
		Country: Japan
		Sample size: *n* = 30 (A) M-16V given several hours (mean: 7.2 h) after birth; *n* = 10 (B) M-16V given >24 h (mean: 36.5 h) after birth; *n* = 10 (C) Control fed normally without probiotic supplement; *n* = 10
		Intervention and dose: 1.6 × 10^8^ CFU twice daily in 0.5 mL of 5% glucose sterile water.
		Duration of supplementation: Continued until discharged.
		Main outcomes: (1) Significant increase in bifidobacterial colonisation in both groups A and B. (2) Significant earlier detection of bifidobacteria and a significant decrease in the cell numbers of *Enterobacteriaceae* were observed in group A.
		Study limitations: (1) This was a non-double-blinded randomised controlled trial which may introduce potential bias. (2) The sample size was relatively small. (3) Assessment of outcome could not be blinded; however, that was not expected to introduce a major bias because of the objectivity of some of the outcomes.
Ishizeki et al., 2013 [32]	Non-RCT prospective study	Study population: Low birth weight infants (<33 weeks; BW 1000–2000 g) who were ready for feeds within seven days of birth in three cohorts: (1) Control group: October 1999 to June 2000; *n* = 16 (2) Single-strain M-16V group: December 2000 to June 2001; *n* = 15 (3) Three-strain probiotics mixture group: April 2002 to April 2003; *n* = 13
		Country: Japan
		Sample size: 44
		Intervention and dose: (1) Control group: No probiotics (2) Single strain M-16V group: 5 × 10^8^ CFU/day in 1.5 mL sterile water (3) Three-strain probiotics mixture group: *B. longum* BB536, *B. breve* M-16V and *B. infantis* M-63; 5 × 10^8^ CFU/day of each strain in 1.5 mL sterile water
		Duration of supplementation: Six weeks.
		Main outcomes: (1) Both single-strain M-16V and three-strain probiotics mixture groups significantly increased the detection rates and cell numbers of *Bifidobacterium* in the faeces as compared to the control group. (2) Bifidobacteria proportion was significantly higher in the single-strain M-16V group at weeks one to four and in the three-strain probiotics mixture group at weeks one to six as compared to the control group. (3) The proportion of bifidobacteria in the three-strain probiotics mixture group was significantly higher than that in the single-strain M-16V group at weeks one and six. (4) The detection rates of *Clostridium* and proportions of *Enterobacteriaceae* were significantly lower in both probiotic groups.
		Study limitations: (1) This was a retrospective study that relies on the quality of record keeping. (2) The study was conducted at three different timelines which may introduce potential bias. (3) Outcome evaluation could not be blinded; however, that was not expected to introduce a major bias because of the objective of some of the outcomes.
Akiyama et al., 1994 [62]	Non-RCT	Study population: Preterm LBW infants with mean gestation (range) 32.8 weeks (27.8–37.6 weeks) and BW 1486 g (780–2250 g)
		Country: Japan
		Sample size: *n* = 10 (Probiotics: 5; Control: 5)
		Intervention and dose: 5 × 10^8^ CFU/day of M-16V in 1.0 mL of sterile water
		Duration of supplementation: Continued until eight weeks of age
		Main outcomes: (1) Significant increase in bifidobacterial colonisation in M-16V-supplemented infants.
		Study limitations: (1) This was a non-randomised controlled trial which may introduce potential bias. (2) The sample size was very small. (3) Assessment of outcome could not be blinded; however, that was not expected to introduce a major bias because of the objectivity of the outcome.
Akiyama et al., 1994 [64]	Non-RCT	Study population: Preterm LBW infants with mean gestation (range) 32.8 weeks (27.8–37.6 weeks) and BW 1486 g (780–2250 g)
		Country: Japan
		Sample size: *n* = 10 (Probiotics: 5; Control: 5)
		Intervention and dose: 5 × 10^8^ CFU/day of *B. longum* BB536 in 1.0 mL of sterile water
		Duration of supplementation: Continued until eight weeks of age
		Main outcomes: (1) Significant increase in bifidobacterial colonisation in *B. longum* BB536-supplemented infants. (2) This study compared the results obtained in the previous study using M-16V and revealed that while M-16V colonised the premature gut as early as week two after birth and remain dominant, the administered strain of *B. longum* was not detected from week six after birth.
		Study limitations: (1) This was a non-randomised controlled trial comparing the results from the previous study which may introduce potential bias. (2) The sample size was very small. (3) Assessment of outcome could not be blinded; however, that was not expected to introduce a major bias because of the objectivity of the outcome.
Effect of M-16V on prevention of NEC		
Satoh et al., 2007 [44]	Non-RCT retrospective study	Study population: Preterm VLBW and ELBW infants in two cohorts: (1) Control group: January 1994 to December 1998; *n* = 226 (ELBW: 101, VLBW: 125) (2) M-16V group: January 1999 to December 2003; *n* = 338 (ELBW: 220, VLBW: 118)
		Country: Japan
		Sample size: *n* = 564
		Intervention and dose: 1 × 10^9^ CFU/day in milk or mixed with formula
		Duration of supplementation: Commenced within several hours after birth (mean: 7.2 h) and continued till discharge at 37 weeks
		Main outcomes: (1) Significant reduction in the incidence of Stage 1 NEC and infection. (2) Significant reduction in mortality due to infection. (3) Increased survival to discharge: 64.2% (301–600 g), 94% (601–1000 g), and 97.8% (1001–1500 g)
		Study limitations: (1) This was a retrospective study that relies on the quality of record keeping. (2) The study was conducted at two different timelines which may introduce potential bias. (3) Outcome evaluation could not be blinded; however, that was not expected to introduce a major bias because of the objective of some of the outcomes.
Patole et al., 2016 [43]	Non-RCT retrospective study	Study population: Preterm neonates <34 weeks over two epochs: (1) Before probiotic supplementation: December 2008 to November 2010 (2) After probiotic supplementation: June 2012 to May 2014
		Country: Australia
		Sample size: *n* = 1755 (Epoch 1: 835; Epoch 2: 920)
		Intervention and dose: 3 × 10^9^ CFU/day in 1.5 mL of sterile water or breast milk
		Duration of supplementation: Started when the infant was ready for feeds and continued till 37 weeks corrected gestational age
		Main outcomes: (1) Significant reduction in the incidence of NEC ≥ Stage II in infants supplemented with M-16V. (2) Significant reduction in “NEC ≥ Stage II or all-cause mortality”, late-onset sepsis, and age at full feeds in M-16V group. (3) For the subgroup of neonates <28 weeks, the beneficial effects of M-16V did not reach statistical significance.
		Study limitations: (1) This was a retrospective study that relies on the quality of record keeping. (2) The study was conducted at two different timelines which may introduce potential bias.
Clinical studies related to the potential mechanisms of action of M-16V		
Fuji et al., 2006 [70]	Randomised controlled trial	Study population: Preterm infants with mean gestation and mean BW of (1) Probiotics group: 31.3 ± 3.16 weeks and 1378 ± 365 g (2) Control group: 31.2 ± 1.98 weeks and 1496 ± 245 g
		Country: Japan
		Sample size: *n* = 19 (Probiotics: 11; Control: 8)
		Intervention and dose: 1 × 10^9^ CFU/day twice daily in 0.5 mL of 5% glucose solution
		Duration of supplementation: Commenced within several hours after birth and continued till discharge.
		Main outcomes: (1) Significant increase in the expression of serum TGF-β1 level and expression of TGF-β signalling molecule (Smad3) on day 28 in M-16V group. (2) Serum cytokine levels were not different in the two groups.
		Study limitations: (1) The sample size was small. (2) Assessment of outcome could not be blinded; however, that was not expected to introduce a major bias because of the objectivity of the outcome.
Wang et al., 2007 [71]	Randomised controlled trial	Study population: Preterm LBW, VLBW, and ELBW infants (gestation: 23–36 weeks, BW: 414–2124 g)
		Country: Japan
		Sample size: *n* = 66 (ELBW, <1000 g: *n* =22; VLBW, <1500 g: *n* = 22; LBW, <2500 g: *n* = 22). The infants were divided into two groups: Probiotics and Control, 11 each).
		Intervention and dose: 1.6 × 10^8^ CFU/day twice daily in 0.5 mL of 5% glucose sterile distilled water.
		Duration of supplementation: From birth till discharge
		Main outcomes: (1) Significant increase in the ratio of acetate to total SCFAs in all M-16V-supplemented infants. (2) Significant reduction in faecal butyrate levels in ELBW and VLBW infants supplemented with M-16V.
		Study limitations: (1) The sample size was small. (2) Assessment of outcome could not be blinded; however, that was not expected to introduce a major bias because of the objectivity of the outcome.

BW, birth weight; CFU, colony-forming units; Non-RCT, non-randomised controlled trial; *B. breve*, *Bifidobacterium breve*; *B. infantis*, *Bifidobacterium infantis*; *B. longum*, *Bifidobacterium longum*; SGA, small for gestational age; IUGR, intrauterine growth retardation; LBW, low birth weight; VLBW, very low birth weight; ELBW, extremely low birth weight; NEC, necrotising enterocolitis; SCFAs, short-chain fatty acids; TGF-β, transforming growth factor-beta.

**Table 2:** Summary from clinical studies of the effects of M-16V on allergic disorders.

Reference	Type of Allergy	Study Design	Study Characteristics
Hattori et al., 2003 [112]	Atopic dermatitis (eczema)	Randomised controlled trial	Study population: Infants aged 8.6 ± 4.5 months
			Country: Japan
			Sample size: *n* = 15 (Probiotics: 8; Control: 7)
			Intervention and dose: 5 × 10^9^ CFU/day
			Duration of supplementation: One month
			Main outcomes: (1) Significant increase in the proportion of *Bifidobacterium* in the faecal microflora in M-16V group. (2) Significant reduction in the proportion of total aerobes in M-16V group. (3) Significant improvement in the allergic symptoms (cutaneous symptom score and total allergic score) in M-16V group. (4) No significant correlation between the changes in allergic symptoms and changes in intestinal microflora.
			Study limitations: (1) The sample size was small. (2) Assessment of outcome could not be blinded; however, that was not expected to introduce a major bias because of the objectivity of some of the outcomes.
Taniuchi et al., 2005 [113]	Food allergy	Randomised controlled trial	Study population: Infants aged 3.1–18.5 months with cow’s milk hypersensitivity and atopic dermatitis
			Country: Japan
			Sample size: *n* = 17 (Probiotics: 10; Control: 7)
			Intervention and dose: 5 × 10^9^ CFU/day
			Duration of supplementation: Three months
			Main outcomes: (1) Significant increase in the proportion of *Bifidobacterium* in the faecal microflora in M-16V group. (2) Significant reduction in the proportion of total aerobic bacteria in M-16V group. (3) Significant improvement in the allergic symptoms in M-16V group as compared to the beginning of the study. (4) In the control group, no changes to the overall faecal microflora and total allergic score during the entire study period.
			Study limitations: (1) The sample size was small. (2) Assessment of outcome could not be blinded; however, that was not expected to introduce a major bias because of the objectivity of some of the outcomes. (3) Outcomes were compared with the baseline but not the control group.
Del Giudice et al., 2017 [111]	Allergic rhinitis	Randomised, double-blinded, placebo-controlled	Study population: Children aged 9 ± 2.2 years with pollen-induced IgE-mediated allergic rhinitis and intermittent asthma
			Country: Italy
			Sample size: *n* = 40 (Probiotics: 20; Placebo: 20)
			Intervention and dose: one sachet/day *B. breve* M-16V: 1 × 10^9^ CFU *B. longum* BB536: 3 × 10^9^ CFU *B. infantis* M-63: 1 × 10^9^ CFU
			Duration of supplementation: Four weeks
			Main outcomes: (1) Significant improvement of allergic symptoms and quality of life in children treated with the probiotics mixture. (2) The intergroup analysis showed that probiotics mixture was significantly more superior to the placebo for all parameters.
			Study limitations: The sample size was relatively small.
Enomoto et al., 2014 [114]	Atopic dermatitis (eczema)	Non-RCT open trial	Study population: Mother–infant pairs; maternal age: (1) Probiotics group: 22–41 years; (2) Control group: 21–38 years.
			Country: Japan
			Sample size: *n* = 166 (Probiotics: 130; Control: 36)
			Intervention and dose: Pregnant women: 2 sachets/day; infants: 1 sachet/day *B. breve* M-16V: 5 × 10^9^ CFU *B. longum* BB536: 5 × 10^9^ CFU
			Duration of supplementation: One month before the expected date of delivery and postnatally to the infants for six months.
			Main outcomes: (1) Significant reduction in the risk of developing eczema/atopic dermatitis during the first 18 months of life in the probiotics group. (2) The proportion of Proteobacteria was significantly lower in mothers at the time of delivery who received probiotics supplementation when compared with the control group and was positively correlated with that of infants at four months of age. (3) No adverse effects were related to the use of probiotics.
			Study limitations: (1) This was a non-randomised trial which may introduce potential bias. (2) Assessment of outcome could not be blinded; however, that was not expected to introduce a major bias because of the objectivity of some of the outcomes.
Van der Aa et al., 2011 [116]	Atopic dermatitis (eczema)	Double-blinded, placebo-controlled multicentre trial	Study population: Full-term infants aged <7 months with atopic dermatitis.
			Country: Netherlands
			Sample size: *n* = 90 (Synbiotics: 46; Placebo: 44)
			Intervention and dose: Synbiotics consisted of *B. breve* M-16V at a dose of 1.3 × 10^9^ CFU/100 mL and a mixture of 90% scGOS and 10% lcFOS (Immunofortis^®^), 0.8 g/100 mL.
			Duration of supplementation: 12 weeks
			Main outcomes: (1) Of the 75 children (mean age 17.3 months) completed the one-year follow-up evaluation, the prevalence of “frequent wheezing” and “wheezing and/or noisy breathing apart from colds” was significantly lower in the synbiotic than in the placebo. (2) Significantly fewer children in the synbiotic than in the placebo group had started to use asthma medication after baseline. (3) Total IgE levels did not differ between the two groups.
			Study limitations: The study tested the effect of M-16V in a synbiotic formulation.

CFU, colony-forming units; IgE, immunoglobulin E; Non-RCT, non-randomised controlled trial; *B. breve*, *Bifidobacterium breve*; *B. infantis*, *Bifidobacterium infantis*; *B. longum*, *Bifidobacterium longum*; scGOS, short-chain galactooligosaccharides; lcFOS, long-chain fructooligosacharides.

## Supplementary Materials

The following are available online at https://www.mdpi.com/2072-6643/11/8/1724/s1, Table S1. Summary of publications related to *Bifidobacterium breve* M-16V; Table S2. Articles related to *Bifidobacterium breve* M-16V.
