# Supplementary material for: Exploring the Science behind Bifidobacterium breve M-16V in Infant Health
Source: Nutrients. 2019 Jul 25;11(8):1724. doi: 10.3390/nu11081724 (PMC6723912; doi:10.3390/nu11081724)
Supplement: Supplementary file 1 [file nutrients-11-01724-s001.pdf]

Table S1. Summary of publications related to *Bifidobacterium breve* M-16V.

Intervention	Study model/subject	Target	Number of studies
Grand total			60
Single strain of M-16V			31
	In vitro/mice/rat	Total	16
		Preterm birth complications	1
		Allergy	3
		Safety/stability	5
		Gut microbiota/others	7
	Infant	Total	14
		Preterm birth complications	11
		Allergy	3
		Safety/stability	0
		Gut microbiota/others	0
	Children/adults/others	Total	1
		Preterm birth complications	0
		Allergy	0
		Safety/stability	0
		Gut microbiota/others	1
Probiotics mixture with other strains			5
	In vitro/mice/rat	Total	0
		Preterm birth complications	0
		Allergy	0
		Safety/stability	0
		Gut microbiota/others	0
	Infant	Total	2
		Preterm birth complications	1
		Allergy	1
		Safety/stability	0
		Gut microbiota/others	0
	Children/adults/others	Total	3
		Preterm birth complications	0
		Allergy	1
		Safety/stability	0
		Gut microbiota/others	2
Synbiotics			24
	In vitro/mice/rat	Total	10
		Preterm birth complications	0
		Allergy	6
		Safety/stability	0
		Gut microbiota/others	4
	Infant	Total	11
		Preterm birth complications	1
		Allergy	8
		Safety/stability	0
		Gut microbiota/others	2
	Children/adults/others	Total	3
		Preterm birth complications	0
		Allergy	2
		Safety/stability	0
		Gut microbiota/others	1

Table S2. Articles related to *Bifidobacterium breve* M-16V

No.	Inter-vention	Study model/ subject	Target	Article	Title	Authors	Publisher	Year	DOI
Articles obtained from database search									
1*	M-16V	Infant	Preterm	Review	Bifidobacterium breve M-16V as a Probiotic for Preterm Infants: A Strain-Specific Systematic Review	Athalye-Jape, Gayatri; Rao, Shripada; Simmer, Karen; Patole, Sanjay	JPEN. Journal of parenteral and enteral nutrition	2018	10.1177/0148607117722749
2	M-16V	Infant	Allergy	Original article	Exploring Immune Development in Infants With Moderate to Severe Atopic Dermatitis	Hulshof, Lies; Overbeek, Saskia A; Wyllie, Anne L; Chu, Mei Ling J N; Bogaert, Debby; de Jager, Wilco; Knippels, Leon M J; Sanders, Elisabeth A M; van Aalderen, Wim M C; Garssen, Johan; Van't Land, Belinda; Sprikkelman, Aline B	Frontiers in immunology	2018	10.3389/fimmu.2018.00630
3	M-16V	Infant	Preterm	Original article	Effect of probiotics on C-reactive protein levels in preterm infants: Secondary analysis of a randomized controlled trial	Agrawal, S; Rao, S; Nathan, E A; Patole, S	Journal of neonatal-perinatal medicine	2018	10.3233/NPM-181763
4	M-16V	Infant	Preterm	Original article	Probiotics and antimicrobial protein and peptide levels in preterm infants	Strunk, Tobias; Hibbert, Julie; Doherty, Dorota; Granland, Caitlyn; Trend, Stephanie; Simmer, Karen; Burgner, David; Patole, Sanjay; Currie, Andrew	Acta paediatrica	2017	10.1111/apa.13826
5	M-16V	Mice	Allergy	Original article	Dietary Intervention with β-Lactoglobulin-Derived Peptides and a Specific Mixture of Fructo-Oligosaccharides and Bifidobacterium breve M-16V Facilitates the Prevention of Whey-Induced Allergy in Mice by Supporting a Tolerance-Prone Immune Environment	Kostadinova, Atanaska I; Pablos-Tanarro, Alba; Diks, Mara A P; van Esch, Betty C A M; Garssen, Johan; Knippels, Léon M J; Willemsen, Linette E M	Frontiers in immunology	2017	10.3389/fimmu.2017.01303
6	M-16V	Rat	Immune	Original article	Differences between live and heat-killed bifidobacteria in the regulation of immune function and the intestinal environment	Sugahara, H; Yao, R; Odamaki, T; Xiao, J Z	Beneficial microbes	2017	10.3920/BM2016.0158

No.	Inter-vention	Study model/ subject	Target	Article	Title	Authors	Publisher	Year	DOI
7*	M-16V	Infant	Preterm	Original article	Effect of Bifidobacterium breve M-16V supplementation on faecal bifidobacteria in growth restricted very preterm infants - analysis from a randomised trial	Patole, Sanjay K; Keil, Anthony D; Nathan, Elizabeth; Doherty, Dorota; Esvaran, Meera; Simmer, Karen N; Conway, Patricia	The journal of maternal-fetal & neonatal medicine	2016	10.3109/14767058.2016.1147554.
8	M-16V	Rat	Immune	Original article	Preclinical Immunomodulation by the Probiotic Bifidobacterium breve M-16V in Early Life	Rigo-Adrover, Maria Del Mar; Franch, Àngels; Castell, Margarida; Pérez-Cano, Francisco José	PloS one	2016	10.1371/journal.pone.0166082
9	M-16V	Children	IBS	Review	Probiotics for Irritable Bowel Syndrome: Clinical Data in Children	Giannetti, Eleonora; Staiano, Annamaria	Journal of pediatric gastroenterology and nutrition	2016	10.1097/MPG.0000000000001220
10*	M-16V	Infant	Preterm	Original article	Benefits of Bifidobacterium breve M-16V Supplementation in Preterm Neonates - A Retrospective Cohort Study	Patole, Sanjay K; Rao, Shripada C; Keil, Anthony D; Nathan, Elizabeth A; Doherty, Dorota A; Simmer, Karen N	PloS one	2016	10.1371/journal.pone.0150775
11*	M-16V	Rat	Preterm	Original article	Bifidobacterium breve prevents necrotising enterocolitis by suppressing inflammatory responses in a preterm rat model	Satoh, T; Izumi, H; Iwabuchi, N; Odamaki, T; Namba, K; Abe, F; Xiao, J Z	Beneficial microbes	2016	10.3920/BM2015.0035
12*	M-16V	Rat	DSS	Original article	Bifidobacterium breve alters immune function and ameliorates DSS-induced inflammation in weanling rats	Izumi, Hirohisa; Minegishi, Mario; Sato, Yohei; Shimizu, Takashi; Sekine, Kazunori; Takase, Mitsunori	Pediatric research	2015	10.1038/pr.2015.115
13*	M-16V	Infant	Preterm	Original article	Effect of Bifidobacterium breve M-16V supplementation on fecal bifidobacteria in preterm neonates--a randomised double blind placebo controlled trial	Patole, Sanjay; Keil, Anthony D; Chang, Annie; Nathan, Elizabeth; Doherty, Dorota; Simmer, Karen; Esvaran, Meera; Conway, Patricia	PloS one	2014	10.1371/journal.pone.0089511
14*	M-16V	*In vitro*	Immune	Original article	Immunoregulatory effect of bifidobacteria strains in porcine intestinal epithelial cells through modulation of ubiquitin-editing enzyme A20 expression	Tomosada, Yohsuke; Villena, Julio; Murata, Kozue; Chiba, Eriko; Shimazu, Tomoyuki; Aso, Hisashi; Iwabuchi, Noriyuki; Xiao, Jin-zhong; Saito, Tadao; Kitazawa, Haruki	PloS one	2013	10.1371/journal.pone.0059259

No.	Inter-vention	Study model/ subject	Target	Article	Title	Authors	Publisher	Year	DOI
15*	M-16V	Rat	Immune	Original article	Effects of Bifidobacterium breve on inflammatory gene expression in neonatal and weaning rat intestine	Ohtsuka, Yoshikazu; Ikegami, Takako; Izumi, Hirohisa; Namura, Mariko; Ikeda, Tomomi; Ikuse, Tamaki; Baba, Yosuke; Kudo, Takahiro; Suzuki, Ryuyo; Shimizu, Toshiaki	Pediatric research	2012	10.1038/pr.2011.11
16	M-16V	*In vitro*	Immune	Original article	Bifidobacterium breve - HT-29 cell line interaction: modulation of TNF-α induced gene expression	Boesten, R J; Schuren, F H J; Willemsen, L E M; Vriesema, A; Knol, J; De Vos, W M	Beneficial microbes	2011	10.3920/BM2011.0005
17	M-16V	Infant	Preterm	Original article	Beneficial microbes for premature infants, and children with malignancy undergoing chemotherapy	Yamashiro, Y; Nagata, S	Beneficial microbes	2010	10.3920/BM2010.0035
18*	M-16V	Mice	Safety	Original article	Safety evaluation of probiotic bifidobacteria by analysis of mucin degradation activity and translocation ability	Abe, Fumiaki; Muto, Masamichi; Yaeshima, Tomoko; Iwatsuki, Keiji; Aihara, Hiroaki; Ohashi, Yuji; Fujisawa, Tomohiko	Anaerobe	2010	10.1016/j.anaerobe.2009.07.006
19*	M-16V	Mice	Allergy	Original article	Oral treatment with probiotics reduces allergic symptoms in ovalbumin-sensitized mice: a bacterial strain comparative study	Hougee, S; Vriesema, A J M; Wijering, S C; Knippels, L M J; Folkerts, G; Nijkamp, F P; Knol, J; Garssen, J	International archives of allergy and immunology	2010	10.1159/000236000
20*	M-16V	Rat	Allergy	Original article	Suppressive effects of bifidobacterium breve strain M-16V on T-helper type 2 immune responses in a murine model	Inoue, Yumi; Iwabuchi, Noriyuki; Xiao, Jin-Zhong; Yaeshima, Tomoko; Iwatsuki, Keiji	Biological & pharmaceutical bulletin	2009	10.1248/bpb.32.760
21*	M-16V	Infant	Preterm	Original article	Effects of oral administration of bifidobacterium breve on fecal lactic acid and short-chain fatty acids in low birth weight infants	Wang, Chongxin; Shoji, Hiromichi; Sato, Hiroaki; Nagata, Satoru; Ohtsuka, Yoshikazu; Shimizu, Toshiaki; Yamashiro, Yuichiro	Journal of pediatric gastroenterology and nutrition	2007	10.1097/01.mpg.0000252184.89922.5f
22*	M-16V	Infant	Allergy	Original article	Administration of Bifidobacterium to infants with atopic dermatitis: Changes in fecal microflora and clinical symptoms	Taniuchi, Shoichiro; Hattori, Kazuhiro; Yamamoto, Akemi; Sasai, Misa; Hatano, Yasuko; Kojima, Takatsugu; Kobayashi, Yohnosuke; Iwamoto, Hiroshi; Yaeshima, Tomoko	Journal of Applied Research	2005	-

No.	Inter-vention	Study model/ subject	Target	Article	Title	Authors	Publisher	Year	DOI
23*	M-16V	Infant	Allergy	Original article	Effects of administration of bifidobacteria on fecal microflora and clinical symptoms in infants with atopic dermatitis	Hattori, Kazuhiro; Yamamoto, Akemi; Sasai, Misa; Taniuchi, Shoichiro; Kojima, Takatsugu; Kobayashi, Yohnosuke; Iwamoto, Hiroshi; Namba, Kyoko; Yaeshima, Tomoko	Arerugi = [Allergy]	2003	-
24*	M-16V	*In vitro*	Safety	Original article	Probiotic characteristics and in vitro compatibility of a combination of Bifidobacterium breve M-16 V, Bifidobacterium longum subsp. infantis M-63 and Bifidobacterium longum subsp. longum BB536	Marco Toscano & Elena De Vecchi & Arianna Gabrieli & Gian Vincenzo Zuccotti & Lorenzo Drago	Annals of Microbiology	2015	10.1007/s13213-014-0953-5
25*	M-16V	Mice	Immune	Original article	Immunobiotic Lactobacillus jensenii elicits anti-inflammatory activity in porcine intestinal epithelial cells by modulating negative regulators of the Toll-like receptor signaling pathway.	Shimazu T, Villena J, Tohno M, Fujie H, Hosoya S, Shimosato T, Aso H, Suda Y, Kawai Y, Saito T, Makino S, Ikegami S, Itoh H, Kitazawa H.	Infect Immun.	2012	10.1128/IAI.05729-11
26*	M-16V	*In vitro*	Safety	Original article	Antibiotic susceptibility of bifidobacterial strains distributed in the Japanese market	Xiao JZ, Takahashi S, Odamaki T, Yaeshima T, Iwatsuki K.	Biosci Biotechnol Biochem.	2010	10.1271/bbb.90659
27	M-16V	Infant	Preterm	Original article	Probiotic Supplementation Dramatically Decreases Infection Deaths in Extremely and Very Low Birth Weight Infants	Hiroaki Sato, Masaki Daigo, Kazuhiro Ohzeki, Hikaru Umezaki, Ayako Kudo, Yuichiro Yamashiro	Juntendo Medical Journal	2009	10.14789/pjmj.55.136
28*	M-16V	Rat	Safety	Original article	Safety Evaluation of Two Probiotic Bifidobacterial Strains, Bifidobacterium breve M-16V and Bifidobacterium infantis M-63, by Oral Toxicity Tests Using Rats	Fumiaki Abe, Tomoko Yaeshima, Keiji Iwatsuki	Bioscience and Microflora	2009	10.12938/bifidus.28.7
29*	M-16V	*In vitro*	Stability	Original article	Stability of bifidobacteria in powdered formula	Fumiaki Abe Hirofumi Miyauchi Ayako Uchijima Tomoko Yaeshima Keiji Iwatsuki	International Journal of Food Science and Technology	2009	10.1111/j.1365-2621.2008.01881.x

No.	Inter-vention	Study model/ subject	Target	Article	Title	Authors	Publisher	Year	DOI
30*	M-16V	Infant	Preterm	Original article	Bifidobacterium breve enhances transforming growth factor beta1 signaling by regulating Smad7 expression in preterm infants	Fujii T, Ohtsuka Y, Lee T, Kudo T, Shoji H, Sato H, Nagata S, Shimizu T, Yamashiro Y.	J Pediatr Gastroenterol Nutr.	2006	10.1097/01.mpg.0000228100.04702.f8
31*	M-16V	Infant	Preterm	Original article	Effects of bifidobacterium breve supplementation on intestinal flora of low birth weight infants	Li Y, Shimizu T, Hosaka A, Kaneko N, Ohtsuka Y, Yamashiro Y.	Pediatr Int.	2004	10.1111/j.1442-200x.2004.01953.x
32*	M-16V/ Probiotics mixture	Infant	Preterm	Original article	Effect of administration of bifidobacteria on intestinal microbiota in low-birth-weight infants and transition of administered bifidobacteria: a comparison between one-species and three-species administration	Ishizeki, Shinobu; Sugita, Masaoki; Takata, Masaaki; Yaeshima, Tomoko	Anaerobe	2013	10.1016/j.anaerobe.2013.08.002
33	Probiotics mixture	Elderly	Cognitive function	Original article	Effect of combined bifidobacteria supplementation and resistance training on cognitive function, body composition and bowel habits of healthy elderly subjects	Inoue, T; Kobayashi, Y; Mori, N; Sakagawa, M; Xiao, J-Z; Moritani, T; Sakane, N; Nagai, N	Beneficial microbes	2018	10.3920/BM2017.0193
34*	Probiotics mixture	Children	Allergy	Original article	Bifidobacterium mixture (B longum BB536, B infantis M-63, B breve M-16V) treatment in children with seasonal allergic rhinitis and intermittent asthma	Miraglia Del Giudice, Michele; Indolfi, Cristiana; Capasso, Michele; Maiello, Nunzia; Decimo, Fabio; Ciprandi, Giorgio	Italian journal of pediatrics	2017	10.1186/s13052-017-0340-5
35	Probiotics mixture	Children	Abdominal pain & QOL	Original article	A Mixture of 3 Bifidobacteria Decreases Abdominal Pain and Improves the Quality of Life in Children With Irritable Bowel Syndrome: A Multicenter, Randomized, Double-Blind, Placebo-Controlled, Crossover Trial	Giannetti, Eleonora; Maglione, Marco; Alessandrella, Annalisa; Strisciuglio, Caterina; De Giovanni, Donatella; Campanozzi, Angelo; Miele, Erasmo; Staiano, Annamaria	Journal of clinical gastroenterology	2017	10.1097/MCG.0000000000000528
36*	Probiotics mixture	Infant	Allergy	Original article	Effects of bifidobacterial supplementation to pregnant women and infants in the prevention of allergy development in infants and on fecal microbiota	Enomoto, Tadao; Sowa, Masanori; Nishimori, Keiji; Shimazu, Shinichiro; Yoshida, Akira; Yamada, Kazuko; Furukawa, Fukumi; Nakagawa, Takemasa; Yanagisawa, Naotake; Iwabuchi, Noriyuki; Odamaki, Toshitaka; Abe, Fumiaki; Nakayama, Jiro; Xiao, Jin-Zhong	Allergology international : official journal of the Japanese Society of Allergology	2014	10.2332/allergolint.13-OA-0683

No.	Inter-vention	Study model/ subject	Target	Article	Title	Authors	Publisher	Year	DOI
37	Synbiotics	Infant	Allergy	Original article	A specific synbiotic-containing amino acid-based formula restores gut microbiota in non-IgE mediated cow's milk allergic infants: a randomized controlled trial	Wopereis, Harm; van Ampting, Marleen T J; Cetinyurek-Yavuz, Aysun; Slump, Rob; Candy, David C A; Butt, Assad M; Peroni, Diego G; Vandenplas, Yvan; Fox, Adam T; Shah, Neil; Roeselers, Guus; Harthoorn, Lucien F; Michaelis, Louise J; Knol, Jan; West, Christina E	Clinical and translational allergy	2019	10.1186/s13601-019-0267-6
38	Synbiotics	Rat	Immune	Original article	The Combination of Bifidobacterium breve and Three Prebiotic Oligosaccharides Modifies Gut Immune and Endocrine Functions in Neonatal Mice	Izumi, Hirohisa; Ehara, Tatsuya; Sugahara, Hirosuke; Matsubara, Takeshi; Mitsuyama, Eri; Nakazato, Yuki; Tsuda, Muneya; Shimizu, Takashi; Odamaki, Toshitaka; Xiao, Jin-Zhong; Takeda, Yasuhiro	The Journal of nutrition	2019	10.1093/jn/nxy248
39	Synbiotics	Infant	Allergy	Original article	A specific synbiotic-containing amino acid-based formula in dietary management of cow's milk allergy: a randomized controlled trial	Fox, Adam T; Wopereis, Harm; Van Ampting, Marleen T J; Oude Nijhuis, Manon M; Butt, Assad M; Peroni, Diego G; Vandenplas, Yvan; Candy, David C A; Shah, Neil; West, Christina E; Garssen, Johan; Harthoorn, Lucien F; Knol, Jan; Michaelis, Louise J	Clinical and translational allergy	2019	10.1186/s13601-019-0241-3
40	Synbiotics	Mice	Obesity	Original article	Specific synbiotics in early life protect against diet-induced obesity in adult mice	Mona Mischke, Tulika Arora, Sebastian Tims, Eefje Engels, Nina Sommer, Kees van Limpt, Annemarie Baars, Raish Oozeer, Annemarie Oosting, Fredrik Bäckhed, Jan Knol	Diabetes Obesity & Metabolism	2018	10.1111/dom.13240
41	Synbiotics	Infant	Preterm	Original article	Outcomes in preterm small versus appropriate for gestation infants after Bifidobacterium breve M-16 V supplementation	Athalye-Jape, Gayatri; Minaee, Novia; Nathan, Elizabeth; Simmer, Karen; Patole, Sanjay	J Matern Fetal Neonatal Med	2018	10.1080/14767058.2018.1543657

No.	Inter-vention	Study model/ subject	Target	Article	Title	Authors	Publisher	Year	DOI
42	Synbiotics	Children	Microbiota	Original article	A synbiotic mixture of scGOS/lcFOS and Bifidobacterium breve M-16V increases faecal Bifidobacterium in healthy young children	Kosuwon, P; Lao-Araya, M; Uthaisangsook, S; Lay, C; Bindels, J; Knol, J; Chatchatee, P	Beneficial microbes	2018	10.3920/BM2017.0110
43	Synbiotics	Mice	Rotavirus infections	Original article	Preventive Effect of a Synbiotic Combination of Galacto- and Fructooligosaccharides Mixture With Bifidobacterium breve M-16V in a Model of Multiple Rotavirus Infections	Rigo-Adrover, Maria Del Mar; van Limpt, Kees; Knipping, Karen; Garssen, Johan; Knol, Jan; Costabile, Adele; Franch, Àngels; Castell, Margarida; Pérez-Cano, Francisco José	Frontiers in immunology	2018	10.3389/fimmu.2018.01318
44	Synbiotics	Infant	Allergy	Original article	A synbiotic-containing amino-acid-based formula improves gut microbiota in non-IgE-mediated allergic infants	Candy, David C A; Van Ampting, Marleen T J; Oude Nijhuis, Manon M; Wopereis, Harm; Butt, Assad M; Peroni, Diego G; Vandenplas, Yvan; Fox, Adam T; Shah, Neil; West, Christina E; Garssen, Johan; Harthoorn, Lucien F; Knol, Jan; Michaelis, Louise J	Pediatric research	2017	10.1038/pr.2017.270
45	Synbiotics	Mice	Allergy	Original article	Dietary, nondigestible oligosaccharides and Bifidobacterium breve M-16V suppress allergic inflammation in intestine via targeting dendritic cell maturation	de Kivit, Sander; Kostadinova, Atanaska I; Kerperien, JoAnn; Morgan, Mary E; Muruzabal, Veronica Ayechu; Hofman, Gerard A; Knippels, Leon M J; Kraneveld, Aletta D; Garssen, Johan; Willemsen, Linette E M	Journal of leukocyte biology	2017	10.1189/jlb.3A0516-236R
46	Synbiotics	Infant	Microbiota	Original article	Effect of Synbiotic on the Gut Microbiota of Cesarean Delivered Infants: A Randomized, Double-blind, Multicenter Study	Chua, Mei Chin; Ben-Amor, Kaouther; Lay, Christophe; Neo, Anne G E; Chiang, Wei Chin; Rao, Rajeshwar; Chew, Charmaine; Chaithongwongwatthana, Surasith; Khemapech, Nipon; Knol, Jan; Chongsrisawat, Voranush	Journal of pediatric gastroenterology and nutrition	2017	10.1097/MPG.0000000000001623
47	Synbiotics	Rat	Rotavirus infections	Original article	A combination of scGOS/lcFOS with Bifidobacterium breve M-16V protects suckling rats from rotavirus gastroenteritis	Rigo-Adrover, M; Saldaña-Ruíz, S; van Limpt, K; Knipping, K; Garssen, J; Knol, J; Franch, A; Castell, M; Pérez-Cano, F J	European journal of nutrition	2017	10.1007/s00394-016-1213-1

No.	Inter-vention	Study model/ subject	Target	Article	Title	Authors	Publisher	Year	DOI
48	Synbiotics	Mice	Allergy	Original article	A Specific Mixture of Fructo-Oligosaccharides and Bifidobacterium breve M-16V Facilitates Partial Non-Responsiveness to Whey Protein in Mice Orally Exposed to β-Lactoglobulin-Derived Peptides	Kostadinova, Atanaska I; Meulenbroek, Laura A P M; van Esch, Betty C A M; Hofman, Gerard A; Garssen, Johan; Willemsen, Linette E M; Knippels, Léon M J	Frontiers in immunology	2017	10.3389/fimmu.2016.00673
49	Synbiotics	Infant	Growth	Original article	Infant formula containing galacto-and fructo-oligosaccharides and Bifidobacterium breve M-16V supports adequate growth and tolerance in healthy infants in a randomised, controlled, double-blind, prospective, multicentre study	Abrahamse-Berkeveld, M; Alles, M; Franke-Beckmann, E; Helm, K; Knecht, R; Köllges, R; Sandner, B; Knol, J; Ben Amor, K; Bufe, A	Journal of nutritional science	2016	10.1017/jns.2016.35
50	Synbiotics	Mice	Allergy	Original article	Post-sensitization administration of non-digestible oligosaccharides and Bifidobacterium breve M-16V reduces allergic symptoms in mice	van Esch, Betty C A M; Abbring, Suzanne; Diks, Mara A P; Dingjan, Gemma M; Harthoorn, Lucien F; Vos, A Paul; Garssen, Johan	Immunity, inflammation and disease	2016	10.1002/iid3.101
51	Synbiotics	Mice	Allergy	Original article	The development of allergic inflammation in a murine house dust mite asthma model is suppressed by synbiotic mixtures of non-digestible oligosaccharides and Bifidobacterium breve M-16V	Verheijden, K A T; Willemsen, L E M; Braber, S; Leusink-Muis, T; Jeurink, P V; Garssen, J; Kraneveld, A D; Folkerts, G	European journal of nutrition	2016	10.1007/s00394-015-0928-8
52	Synbiotics	Infant	Allergy	Original article	Synbiotics-supplemented amino acid-based formula supports adequate growth in cow's milk allergic infants	Burks, A Wesley; Harthoorn, Lucien F; Van Ampting, Marleen T J; Oude Nijhuis, Manon M; Langford, Jane E; Wopereis, Harm; Goldberg, Steven B; Ong, Peck Y; Essink, Brandon J; Scott, Robert B; Harvey, Bryan M	Pediatric allergy and immunology	2015	10.1111/pai.12390
53	Synbiotics	Human	Allergy	Original article	Novel immunotherapy approaches to food allergy	Hayen, Simone M; Kostadinova, Atanaska I; Garssen, Johan; Otten, Henny G; Willemsen, Linette E M	Current opinion in allergy and clinical immunology	2014	10.1097/ACI.0000000000000109

No.	Inter-vention	Study model/ subject	Target	Article	Title	Authors	Publisher	Year	DOI
54	Synbiotics	Infant	Allergy	Original article	Intestinal epithelium-derived galectin-9 is involved in the immunomodulating effects of nondigestible oligosaccharides	de Kivit, Sander; Kraneveld, Aletta D; Knippels, Leon M J; van Kooyk, Yvette; Garssen, Johan; Willemsen, Linette E M	Journal of innate immunity	2013	10.1159/000350515
55*	Synbiotics	Infant	Allergy	Original article	No detectable beneficial systemic immunomodulatory effects of a specific synbiotic mixture in infants with atopic dermatitis	van der Aa, L B; Lutter, R; Heymans, H S A; Smids, B S; Dekker, T; van Aalderen, W M C; Sillevis Smitt, J H; Knippels, L M J; Garssen, J; Nauta, A J; Sprikkelman, A B	Clinical and experimental allergy	2012	10.1111/j.1365-2222.2011.03890.x
56	Synbiotics	Human/ Rat	Allergy	Original article	Galectin-9 induced by dietary synbiotics is involved in suppression of allergic symptoms in mice and humans	de Kivit, S; Saeland, E; Kraneveld, A D; van de Kant, H J G; Schouten, B; van Esch, B C A M; Knol, J; Sprikkelman, A B; van der Aa, L B; Knippels, L M J; Garssen, J; van Kooyk, Y; Willemsen, L E M	Allergy	2012	10.1111/j.1398-9995.2011.02771.x
57*	Synbiotics	Infant	Allergy	Original article	Synbiotics prevent asthma-like symptoms in infants with atopic dermatitis	van der Aa, L B; van Aalderen, W M C; Heymans, H S A; Henk Sillevis Smitt, J; Nauta, A J; Knippels, L M J; Ben Amor, K; Sprikkelman, A B	Allergy	2011	10.1111/j.1398-9995.2010.02416.x
58	Synbiotics	Adult	Allergy	Original article	Synbiotics reduce allergen-induced T-helper 2 response and improve peak expiratory flow in allergic asthmatics	van de Pol, M A; Lutter, R; Smids, B S; Weersink, E J M; van der Zee, J S	Allergy	2011	10.1111/j.1398-9995.2010.02454.x
59	Synbiotics	Infant	Allergy	Original article	Effect of a new synbiotic mixture on atopic dermatitis in infants: a randomized-controlled trial	van der Aa, L B; Heymans, H S; van Aalderen, W M; Sillevis Smitt, J H; Knol, J; Ben Amor, K; Goossens, D A; Sprikkelman, A B	Clinical and experimental allergy	2010	10.1111/j.1365-2222.2010.03465.x
60	Synbiotics	Mice	Allergy	Original article	Cow milk allergy symptoms are reduced in mice fed dietary synbiotics during oral sensitization with whey	Schouten, Bastiaan; van Esch, Betty C A M; Hofman, Gerard A; van Doorn, Suzan A C M; Knol, Jan; Nauta, Alma J; Garssen, Johan; Willemsen, Linette E M; Knippels, Léon M J	The Journal of nutrition	2009	10.3945/jn.109.108514

No.	Inter-vention	Study model/ subject	Target	Article	Title	Authors	Publisher	Year	DOI
Additional articles not deposited in database									
61*	M-16V	Infant	Microbiota	Original article	Effects of oral administration of Bifidobacterium breve on development of intestinal microflora in extremely premature infants.	Akiyama, K.; Hosono, S.; Takahashi, E.; Ishizeki, S.; Takigawa, I.; Imura, S.; Yamauchi, K.; Yaeshima, T.; Hayasawa, H.; Shimamura, S.	Acta Neonatol Japonica	1994	-
62*	*B. longum* BB536 (compare with M-16V)	Infant	Microbiota	Original article	Effects of administration of Bifidobacterium in extremely premature infants. Development of intestinal microflora by orally administered Bifidobacterium longum (in comparison with Bifidobacterium breve)	Akiyama, K.; Shimada, M.; Ishizeki, S.; Takigawa, I.; Imura, S.; Yamauchi, K.; Hatano, M.; Abe, N.; Yaeshima, T.; Hayasawa, H.	Acta Neonatol Japonica	1994	-
63*	M-16V	*In vitro*	Immune	Original article	Transforming growth factor-β: an important cytokine in the mucosal immune response.	Ohtsuka, Y.; Sanderson, I.R.	Curr Opin Gastroenterol	2000	https://www.ncbi.nlm.nih.gov/pubmed/17031135
64*	M-16V	Infant	Preterm	Original article	Bifidobacteria prevents necrotizing enterocolitis and infection in preterm infants.	Satoh, Y.; Shinohara, K.; Umezaki, H.; Shoji, H.; Satoh, H.; Ohtsuka, Y.; Shiga, S.; Nagata, S.; Shimizu, T.; Yamashiro, Y.	Int J Probiotics Prebiotics	2007	http://www.nchpjournals.com/manuscript/uploads/article_727.pdf

*Article numbers with asterisk were selected and included in this review.
